# SSR markers in transcripts of genes linked to post-transcriptional and transcriptional regulatory functions during vegetative and reproductive development of *Elaeis guineensis*

**DOI:** 10.1186/1471-2229-12-1

**Published:** 2012-01-03

**Authors:** Timothy John Tranbarger, Wanwisa Kluabmongkol, Duangjai Sangsrakru, Fabienne Morcillo, James W Tregear, Somvong Tragoonrung, Norbert Billotte

**Affiliations:** 1 IRD, UMR DIADE (IRD, UM2), 911 Avenue Agropolis BP 64501, 34394, Montpellier cedex 5, France; 2 Genome Institute, National Center for Genetic Engineering and Biotechnology (BIOTEC), 113 Thailand Science Park, Phahonyothin Road, Klong 1, Klong Luang, Pathumthani 12120, Thailand; 3 CIRAD, UMR DIADE, F-34394 Montpellier, France; 4 CIRAD, UMR AGAP, F-34398 Montpellier, France

## Abstract

**Background:**

The oil palm (*Elaeis guineensis *Jacq.) is a perennial monocotyledonous tropical crop species that is now the world's number one source of edible vegetable oil, and the richest dietary source of provitamin A. While new elite genotypes from traditional breeding programs provide steady yield increases, the long selection cycle (10-12 years) and the large areas required to cultivate oil palm make genetic improvement slow and labor intensive. Molecular breeding programs have the potential to make significant impacts on the rate of genetic improvement but the limited molecular resources, in particular the lack of molecular markers for agronomic traits of interest, restrict the application of molecular breeding schemes for oil palm.

**Results:**

In the current study, 6,103 non-redundant ESTs derived from cDNA libraries of developing vegetative and reproductive tissues were annotated and searched for simple sequence repeats (SSRs). Primer pairs from sequences flanking 289 EST-SSRs were tested to detect polymorphisms in elite breeding parents and their crosses. 230 of these amplified PCR products, 88 of which were polymorphic within the breeding material tested. A detailed analysis and annotation of the EST-SSRs revealed the locations of the polymorphisms within the transcripts, and that the main functional category was related to transcription and post-transcriptional regulation. Indeed, SSR polymorphisms were found in sequences encoding AP2-like, bZIP, zinc finger, MADS-box, and NAC-like transcription factors in addition to other transcriptional regulatory proteins and several RNA interacting proteins.

**Conclusions:**

The identification of new EST-SSRs that detect polymorphisms in elite breeding material provides tools for molecular breeding strategies. The identification of SSRs within transcripts, in particular those that encode proteins involved in transcriptional and post-transcriptional regulation, will allow insight into the functional roles of these proteins by studying the phenotypic traits that cosegregate with these markers. Finally, the oil palm EST-SSRs derived from vegetative and reproductive development will be useful for studies on the evolution of the functional diversity within the palm family.

## Background

Oil palm (*Elaeis guineensis *Jacq.), a perennial monocotyledonous tropical crop species that belongs to the family Arecaceae, is now the world's number one source of edible vegetable oil, and also the richest dietary source of provitamin A. While the worldwide demand for palm oil increases each year, new elite genotypes from traditional breeding programs provide a yield increase of only 1% per year and the long selection cycle (10-12 years) makes genetic improvement slow [1]. Furthermore, to increase overall oil productivity without new expansion of oil palm cultivation in tropical forest regions with high biodiversity, there is a great need to develop molecular markers for molecular assisted breeding programs targeted to facilitate genetic improvement in yield, and as markers of other important agronomic characters of interest.

Microsatellite markers or simple sequence repeats (SSRs) are tandem DNA repeats from 1-6 bp that are found throughout the coding and non-coding regions of eukaryotic genomes. Non-coding SSRs are often highly polymorphic, co-dominant and simple to detect, and therefore easily adapted to use in high-throughput PCR-based genotyping. They have also been developed for a wide number of crop species and used for various important applications such as genome mapping, diversity studies, and QTL analysis [2]. Due to their highly polymorphic nature, non-coding SSRs are especially useful for fingerprinting or varietal identification studies, but have limited use for studies with more distantly related species [3]. While SSRs derived from non-coding genomic DNA are not transcribed, SSRs identified within transcript sequences can be associated to a function and linked more easily to a phenotypic trait of interest, making them useful for functional diversity studies [2,3]. In addition, ESTs within genic regions are more transferable for use in diversity studies with more distantly related species. Furthermore, the presence of SSRs in transcribed regions can result in changes in function, transcription or translation. Indeed, SSRs in the coding regions that result in amino acid changes can cause either gain or loss of function, while the presence of SSRs in the 5'UTR can affect transcription or translation, and SSRs in the 3'UTR can affect splicing [3-5].

In the case of oil palm, previous studies have reported the identification of putative SSRs within available EST data [6-8]. However, very few EST-SSRs have been tested nor their usefulness been compared with SSRs identified from the non-coding parts of the genome [9,10]. Indeed, the genetic maps available for oil palm are mainly based on anonymous non-coding SSRs, AFLPs or RAPDs [9,11-14]. The oil palm EST-SSRs identified thus far that reveal polymorphisms are mainly from genes that lack similarity with known sequences or encode proteins with unknown function [7,8]. In fact, in these two recently published articles from oil palm, only two EST-SSRs reported were similar to sequences with known functions. Furthermore, the RNAs used to produce the ESTs for those SSR searches were derived from a narrow range of tissue sources, mainly *in vitro *materials [6-8]. Despite the relatively low number of EST-SSR markers developed for oil palm, the few that have been tested for interspecies transferability indicate great promise for utilization for comparative genomic studies [7,8]. Therefore, a strategy to identify EST-SSRs is not only important for diversity studies as a basis for molecular breeding strategies with oil palm, but in addition, markers in conserved coding regions allow easy transferability to other species within the Arecaceae family and provide tools for evolutionary and functional diversity studies [3-5].

The current study has the objective to identify SSRs in ESTs derived from oil palm and to evaluate their utility as molecular markers with plant material used in genetic improvement programs. In particular, we focused on the identification of SSRs in ESTs that originate from developing vegetative and reproductive tissues, and examine their potential for use in mapping and molecular breeding, in addition to functional diversity and genomics analyses within the Arecaceae family.

## Results and discussion

### Characteristics of SSRs derived from oil palm ESTs

**Figure 1:**
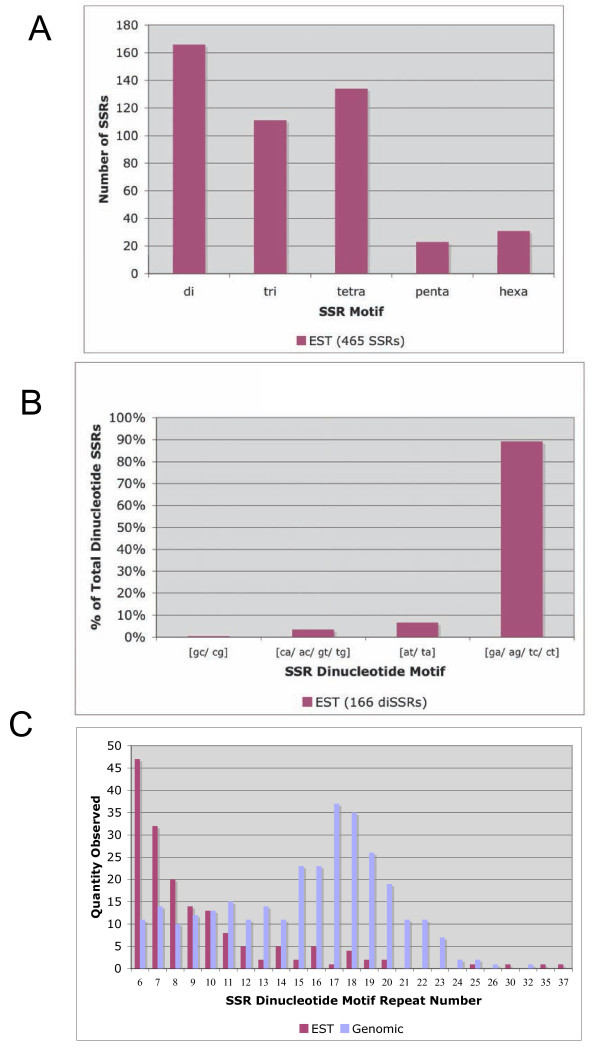
**Characteristics of the EST-SSRs from oil palm identified from vegetative and reproductive development**. **a**. EST-SSR motif densities observed. **b**. Distribution of dinucleotide EST-SSRs observed. **c**. Distribution of dinucleotide repeat numbers observed in ESTs and genomic SSRs.

**Figure 2:**
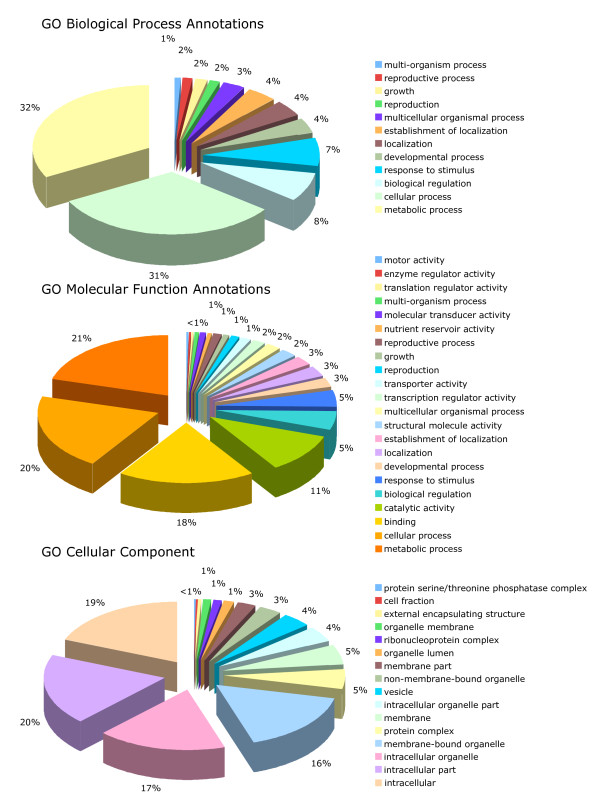
**Gene Ontology annotation of 465 EST-SSRs**. The GO Biological Process, Molecular Function and Cellular Component graphs depict level 2, 2 and 3 annotations respectively.

A total of 12 cDNA libraries constructed from tissues representing different stages of reproductive and vegetative development of the oil palm including the shoot apex, embryogenic cells, somatic and zygotic embryos, male and female inflorescences were analyzed for the presence of SSRs (Table 1) [15-17]. In addition, two of the libraries, including those from the shoot apex (library A1) and from the male inflorescences (M2), were derived from oil palm material that exhibited the mantled abnormality phenotype, [15,16]. From a total of 7,376 redundant ESTs, inter-library cluster analysis resulted in the identification of 6,103 (83%) unigenes comprised of 4,967 singletons and 1136 contigs (Table 2). There were 465 (8% of total unigenes) SSRs found within a total EST sequence of 2,652,262 bp, which corresponds to one SSR for every 5.7 kb of EST sequence. This is higher than the frequencies found previously of 7.7 kb, 8.2 kb and 9.6 kb in oil palm [5,7,8]. In the present study, there were 25 compound SSRs, including 24 doubles and one triple. As previously reported for oil palm the most abundant were those with di (36%) motifs, followed by, tetra (29%), tri (24%), hexa (7%) and penta (5%) motifs (Figure 1a). EST-SSRs with tetra motifs were also abundant in one previous study [6], while very few were observed in other studies [7,8]. The reasons for the discrepancy between the studies are unknown but may be due to differences in the parameters selected for searching for SSRs in the EST sequences. Almost 90% of the dinucleotide SSRs had a ga/ag/tc/ct motif, which confirms what was previously shown for oil palm [6-8] (Figure 1b). Annotation of the SSR-containing ESTs resulted in a total of 538 GO annotations for 264 unigenes, whereas 201 had no similarities to known sequences in the public databases (Table 2). Annotation with GO terms revealed that the ESTs with SSRs were related to a diverse range of putative biological processes, molecular functions and cellular localizations (Figure 2). The largest portions of ESTs were annotated with the GO Biological Process Annotations and Molecular Function Annotations for metabolic (32% and 21% respectively) and cellular (31% and 20% respectively) processes mostly localized intracellularly (19%) within intracellular particles (17%) or organelles (16%).

**Table 1:** Summary of intra-library analysis of ESTs derived from oil palm developmental related cDNA libraries used for the SSR analysis

cDNA Library Source Material	Library code	Valid ESTs	Singletons	Contigs	Unigenes	References
Shoot apex (normal)	A0	313	293	11	304	Jouannic et al. 2005

Shoot apex (abnormal^1^)	A1	998	807	83	890	Jouannic et al. 2005

Early somatic embryos^2 ^(SSH) BAP^3^/no BAP	E1	939	807	66	873	Unpublished

Early somatic embryos^2 ^(SSH) no BAP/BAP	E2	181	179	2	181	Unpublished

Female inflorescences	FO	349	319	13	332	Jouannic et al. 2005

Male inflorescences	MO	625	548	38	586	Jouannic et al. 2005

Male inflorescences SSH normal/abnormal^1^	M1	717	520	77	597	Beule et al. 2011

Male inflorescences SSH abnormal^1^/normal	M2	877	706	60	766	Beule et al. 2011

embryogenic suspension cells SSH +2,4-D^4^	S3	918	717	97	814	Lin et al. 2009

embryogenic suspension cells SSH -2,4-D^5^	S4	949	762	77	839	Lin et al. 2009

Zygotic Embryos un-normalized	Z0	126	94	11	105	Jouannic et al. 2005

Zygotic Embryos SSH-normalized	Z1	384	310	34	344	Unpublished

Total ESTs analyzed		7376				

^1^Trees exhibited mantled flower/fruit phenotype ^2^Somatic embryos developed on solid media with or without ^3^6-benzylaminopurine for one week were used to make two libraries ^4^Embryogenic cells grown under proliferation and somatic embryogenesis ^5^initiation conditions See references for further details of material

**Table 2:** Results compiled from cluster and SSR analysis of the 12 oil palm cDNA libraries

Steps of Analyses	Totals
ESTs	7376

Singletons	4967

Contigs	1136

Unigenes	6103

SSRs found within unigene set	465

EST-SSRs with similarities found by BLASTX at NCBI	186

EST-SSRs similar to Unknown, predicted or hypothetical protein	78

EST-SSRs with no similarities in NCBI databases	201

GO biological process annotations	163

GO molecular function annotations	179

GO cellular component annotations	196

EST-SSRs with annotations and possible primer pairs identified in flanking sequences	316

EST-SSR primer pairs synthesized and tested for polymorphisms in a cross LM2TxDA10D	289

EST-SSR primer pairs that amplified PCR product	230

From earlier studies, a total of 544 genomic SSRs were identified from a total of 378 unisequences, or 243,943 bp [9,10]. The distribution of the repeat number for SSR dinucleotide motifs found in the genomic SSRs was different between the EST-SSRs and the genomic SSRs (Figure 1c). Indeed, a peak of 6 repeats (the minimum repeat number cut-off parameter used to search for SSRs) was observed for the dinucleotide EST-SSRs, while the genomic dinucleotide SSRs had a distribution peak at 17-18 repeats. A higher quantity of low repeat numbers in the coding versus genomic SSRs may reflect the higher selective pressure of the coding portion compared to the noncoding portion of the genome.

### SSRs that detect polymorphisms within LM2T and DA10D crosses

**Figure 3:**
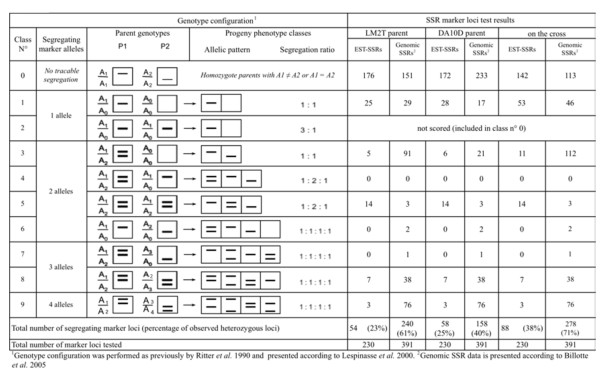
**Genotype configuration and distribution of EST-SSR and genomic SSR marker loci in the LM2T × DA10D cross between two heterozygous parents**.

Of the 465 EST-SSRs found within the unigene set, 316 had possible PCR primer pairs identified and designed in flanking sequences. The LM2T and DA10D parent lineages are currently used for *dura *× *pisifera *crosses in a reciprocal recurrent selection scheme developed for oil palm [18] and also served as the reference cross for the genomic SSR based oil palm genetic map [9]. Therefore, we tested and compared the polymorphisms of 289 EST-SSRs (including all ESTs with annotations) identified in the present study with the polymorphisms revealed by non-coding SSRs described previously [9], using the LM2T and DA10D mapped parents and their progeny. A total of 230 (79%) primer pairs designed from the EST-SSRs (Additional file 1: Table S1) amplified a PCR product while the remainder were either null alleles [19], or simply did not amplify DNA due to incorrect primer design. From these 230 EST-SSR loci, 88 (24%) revealed a polymorphism in the parents (LM2T and/or DA10D) with the 9 expected classes from 1 to 4 segregating SSR alleles in this type of cross between two heterozygous parents (Figure 3). In comparison, from the 391 genomic SSR primer pairs tested previously, there were 278 (71%) loci polymorphic on the same reference cross, with more loci of classes 4 to 9 (43% compared to 27%) heterozygous on both mapped parents. This result suggests that, among the relatively small proportion (7.6%) of SSRs found in unigenes, rather few of these latter, and far less comparatively to genomic SSRs, can actually be mapped on a given genome using SSR markers, at least with the genetic material tested in this study. Therefore, the use of SSRs within EST resources may not be the most efficient method to develop a large number of intragenic markers. However, SSRs remain an important type of polymorphic marker for mapping, in particular for species that lack genome sequence data, and in combination with the range of new polymorphic markers potentially available [20,21].

**Figure 4:**
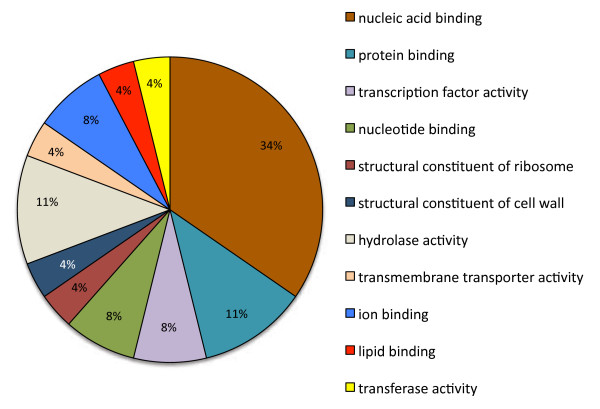
**34 EST-SSRs that detect polymorphisms can be assigned Gene Ontology molecular function annotations**. The graph depicts the level 3 molecular function annotations.

Of the 88 EST-SSR flanking primer pairs that detected a polymorphism, 48 ESTs had no similarities to sequences in available databases, 34 had sequence similarities and could be assigned putative functions based on categories described previously [22], while 6 ESTs were similar to sequences with unknown, unnamed, hypothetical or expressed proteins (Table 3). A detailed annotation of the sequences revealed that the most highly represented functional group (13 of 40, or 32%) was transcription and post-transcriptional regulation, followed by five ESTs with similarities to sequences involved in protein destination and storage, and three involved in signal transduction, cell structure and disease and defense, respectively. Annotation with GO confirmed that the highest proportion of ESTs had functions related to nucleic acid binding, followed by protein binding (Figure 4). Other polymorphisms were found in sequences involved in cell growth and division, cell structure, disease and defense, energy, intracellular traffic, protein synthesis and transport (Table 3).

**Table 3:** List of 40 EST-SSRs with similarities to known genes

Functional Category^1^	GenBank Accession	SSR Locus	SSR Position	SSR Motif^2^	Gene Information^3^	Species	Reference	E-value
Cell Growth/Division	CN599543	mEgEST288	ORF	(acaa)3	Cylicin-2	*Ricinus communis*	ref|XP_002518267.1|	1e-03
	
	GH636902	mEgEST122	5' UTR/ORF	(gtat)3	GASA, gibberelic acid stimulated	*Fagus sylvatica*	emb|CAJ77893.1|	2e-30

Cell Structure	CN601255	mEgEST055	3' UTR	(ga)7	ripening-related protein-like	*Oryza sativa*	dbj|BAD53655.1|	3e-12
	
	GH636141	mEgEST104	5' UTR/ORF	(ga)9	endo-1,3-1,4-beta-d-glucanase	*Elaeis guineensis*	gb|ACF06491.1|	7e-34
	
	GH636466	mEgEST110	5' UTR	(tccc)3	alpha-expansin 6	*Gossypium raimondii*	gb|ABR57477.1|	6e-55

Disease/Defense	CN600757	mEgEST048	3' UTR	(ag)9	metallothionein-like protein	*Typha latifolia*	gb|AAK28022.1|	1e-10
	
	CN601280	mEgEST056	5' UTR	(ct)7	Rubber elongation factor	*Medicago truncatula*	gb|ABD28680.1|	4e-19
	
	GH636774	mEgEST119	3' UTR	(agc)6	fiber protein Fb2	*Zea mays*	ref|NP_001148883.1|	2e-28

Energy	JK669188	mEgEST038	5' UTR	(ct)6	enolase	*Elaeis guineensis*	gb|ACF06525.1|	2e-81

Intracellular traffic	GH636491	mEgEST112	5' UTR	(agg)7	UniGOS12 (GOLGI SNARE 12); SNARE binding	*Arabidopsis thaliana*	ref|NP_182045.1|	1e-37

Protein Destination and Storage	JK669075	mEgEST034	5' UTR	(tc)16	26S proteasome regulatory particle non-ATPase sub-unit 12	*Oryza sativa*	ref|NP_001059500.1|	2e-30
	
	JK669530	mEgEST046	3' UTR	(ag)16	zinc ion binding protein	*Ricinus communis*	ref|XP_002522272.1|	2e-27
	
	JK668742	mEgEST028	ORF	(ga)7	Thioredoxin	*Ricinus communis*	ref|XP_002519481.1|	1e-38
	
	GH635970	mEgEST100	ORF	(tct)11	thioredoxin h	*Hevea brasiliensis*	gb|AAD33596.1|	4e-25
	
	GH636952	mEgEST123	3' UTR	(tgc)5	20S proteasome alpha subunit E	*Glycine max*	sp|Q9M4T8.1|	1e-32

Protein synthesis	GH637071	mEgEST126	3' UTR	(tgg)6	60S ribosomal protein L24	*Elaeis guineensis*	gb|ACF06439.1|	2e-21
	
	GH636588	mEgEST114	ORF	(gga)5	proliferating cell nuclear proteinP120	*Oryza sativa*	dbj|BAD12915.1|	4e-39

Signal Transduction	JK668571	mEgEST023	ORF	(gat)7	rac GTPase activating protein 3	*Lotus japonicus*	gb|AAC62626.1|	3e-19
	
	CN600893	mEgEST053	5' UTR	(tg)7	rac GTPase	*Ricinus communis*	ref|XP_002514274.1|	1e-10
	
	JK668766	mEgEST029	ORF	(gca)5	Calcyclin-binding protein	*Ricinus communis*	ref|XP_002519682.1|	8e-35

Transcription and Post-transcription	CN600270	mEgEST166	ORF	(agc)6	AP2 domain-containing transcription factor	*Musa acuminata*	gb|AAV54598.1|	2e-07
	
	JK668687	mEgEST027	5' UTR	(gtct)4	retinoblastoma-binding protein	*Zea mays*	gb|ABF94566.1|	1e-94
	
	CN601180	mEgEST054	5' UTR	(ct)9	PHD finger family protein	*Arabidopsis lyrata*	ref|XP_002877256.1|	13e-13
	
	GT119420	mEgEST164	ORF	(gcagta)3	bZIP transcription factor	*Oryza sativa*	gb|ACF60482.1|	5e-20
	
	GT119446	mEgEST283	ORF	(aga)6	C3H-related transcription factor	*Oryza sativa*	gb|ACF60482.1|	9e-12
	
	GT120438	mEgEST089	ORF	(ag)9	MADS box transcription factor (AGL2/SEPALLATA) subfamily	*Musa acuminata*	gb|ACJ64678.1|	2e-26
	
	GH637610	mEgEST138	5' UTR	(ga)8	NAM; No apical meristem (NAM) NAC-like protein	*Vitis vinifera*	gb|ACX47024.1|	8e-30
	
	GT119741	mEgEST074	ORF	(cag)5	poly(A)-binding protein	*Nicotiana tabacum*	gb|AAF66825.1|	7e-38
	
	JK669622	mEgEST079	ORF	(aga)5	nucleolar phosphoprotein (RNA binding domain)	*Ricinus communis*	gb|EEF32038.1|	1e-26
	
	GH636728	mEgEST117	ORF	(ag)12	RNA binding (RRM/RBD/RNP motifs) family protein	*Arabidopsis thaliana*	gb|AEE75227.1|	2e-16
	
	GH637298	mEgEST168	ORF	(agac)3	RNA binding protein	*Ricinus communis*	gb|EEF30419.1|	2e-05
	
	CN599492	mEgEST290	ORF	(ctctcc) 4	BCAS2 protein (spliceosome associated protein)	*Zea mays*	ref|NP_001150981.1|	3e-42
	
	JK669619	mEgEST065	3' UTR	(tttttg)3	putative splicing factor 3b, subunit 3 (RNA binding)	*Oryza sativa*	dbj|BAD10377.1|	1e-78

Transporters	JK669486	mEgEST207	3' UTR	(tcaa)3	UniABC transporter family, cholesterol/phospholipid flippase	*Populus trichocarpa*	ref|XP_002308937.1|	5e-09

Unknown or Unclassified Proteins	CN599385	mEgEST002	5' UTR/ORF	(tc)6	predicted protein	*Populus trichocarpa*	ref|XP_002308462.1|	1e-44
	
	CN599993	mEgEST213	ORF	(aga)6	hypothetical protein	*Zea mays*	gb|ABA99397.2|	1e-07
	
	CN600785	mEgEST192	5' UTR	(ct)10	hypothetical protein	*Sorghum bicolor*	ref|XP_002441621.1|	3e-07
	
	CN601056	mEgEST190	ORF	(ccg)8	hypothetical protein	*Vitis vinifera*	ref|XP_002264805.1|	3e-07
	
	CN600741	mEgEST223	ORF	(gag)8	predicted protein	*Hordeum vulgare*	dbj|BAK01617.1|	1e-30
	
	GT120094	mEgEST221	5' UTR	(ttccc)4	hypothetical protein	*Oryza sativa*	ref|NP_001061665.1|	8e-38

Also shown are the SSR primer pairs that detect polymorphisms in reference breeding material and the position of the SSRs within the transcripts

^1^Annotation was assigned to sequences as previously described [16]; ^2^all sequences are in coding sense; ^3^most similar sequence from BLASTX analysis is indicated

An examination of the position of the SSRs within the transcripts revealed that the majority (18) was within the open reading frame (ORF) of the transcript, while eleven were within the 5' untranslated region (UTR), eight within the 3' UTR and 3 overlapped between the 5' UTR start codon and the ORF. Therefore, the majority of the SSRs identified affect the amino acid sequence of the gene product and thus may alter the gene function via a frameshift mutation, while the remainder of the SSRs found in the UTR regions could have an effect on transcription, translation or splicing of gene products [5].

An examination of the distribution of the EST-SSRs found from the vegetative and reproductive libraries used in the present study revealed five EST-SSRs were derived from the apex (A01 and A11), three from the female inflorescence (F01), fourteen from the male inflorescence (M01, M11 and M21), eighteen from the somatic embryo (E11, E21, S31 and S41) libraries, while no SSRs were found in the sequences from the zygotic libraries (Tables 1 and 3). EST-SSR markers that are associated with a given vegetative or reproductive phase may be useful for studies focused on the inter- and intra-specific functional diversity underlying these tissues in the Arecaceae family.

Transcriptional regulation, in particular through the activity of transcription factors, is known to play a central role in the plant growth and development, and during the evolution of plant form [23,24]. A survey of the SSRs in the genome of rice and Arabidopsis indicated that amongst the most common GO categories were those related to the nucleus, transcription factor activity, nucleotide binding and DNA binding [4]. Furthermore, a study with humans also found an enrichment in variable repeats in transcripts involved in transcriptional regulation and development [25]. In the present study, SSR polymorphisms were detected in 6 transcripts that encode proteins similar to those that interact with RNA (spliceosome or RNA binding proteins), five similar to transcription factors (TF) including AP2-like, bZIP, zinc finger, MADS-box, and NAC-like TFs, and two transcriptional regulatory proteins including a PHD finger family protein and a retinoblastoma-binding protein. Interestingly, transcriptional regulators are not only central to the evolution of plant form, but also are associated with domestication of crop species [23,26]. In particular, MADS-box genes were frequent targets of selection during maize domestication [27]. The high number of polymorphisms in transcripts that encode proteins involved in transcriptional regulation in oil palm elite breeding material raises the question of a possible relation to the improvement gained from the reciprocal recurrent selection scheme developed for oil palm. However, the relatively low number of ESTs examined must be taken into account and a conclusive analysis awaits the availability of the genomic sequence of oil palm. Future objectives include the examination of the phenotypic consequences of these SSRs in different oil palm genetic material, and within the Arecaceae family as a whole to determine their relevance to the functional diversity observed.

## Conclusions

SSRs in transcripts encoding proteins involved in transcriptional regulation and other functions found from the current study provide pertinent markers for applications such as mapping, molecular breeding and QTL analysis, in addition to the potential for uses in functional diversity studies within the oil palm and between other palm species. In particular, the identification of SSRs in transcripts related to transcriptional control will allow studies aimed at understanding the functional role of these genes in relation to the emerging domestication of the oil palm. However, it should be noted that due to the limited proportion of polymorphic SSRs present in the coding regions, it is important to develop the full range of other potential polymorphic markers in order to combine structural and functional genomics studies on a large genome-scale to allow marker-assisted selection in oil palm.

## Methods

### Plant material production

The preparation of embryogenic suspension cells and RNA extractions for the suppression subtractive hybridization (SSH) library constructions from the 30-day proliferation cycle and after 16 days of liquid pretreatment to initiate somatic embryogenesis was performed and described previously [17]. In addition, a portion of pretreated embryogenic suspension cells initiated to undergo somatic embryogenesis was plated on solid agar plates containing the basal medium with or without 6-benzylaminopurine (synthetic cytokinin) for further somatic embryo development and collected after 7 days for RNA extractions and SSH library constructions. The material collected for the shoot apex, female and male inflorescences, and zygotic embryos for the unnormalized library constructions was described previously [16]. The material for the normal and abnormal male inflorescences SSH libraries was described and performed previously [15]. The zygotic embryos (3-5.5 months of development) were isolated from *tenera *palm seeds collected from trees (Deli x La Mé origin) cultivated at CRAPP Pobé Station, Benin.

### cDNA library construction

The unnormalized libraries (A0, A1, F0, M0 and Z0) were constructed previously [16] and the SSH libraries were constructed as described previously [17]. The zygotic embryo normalized SSH library was constructed using cDNA made from RNA extracted by RNAeasy lipid (Qiagen) from zygotic embryos (Table 1). The same cDNA was used for both the driver and tester library normalization.

### EST generation, analysis, annotation and data mining to identify SSR markers

The ESTs originating from the SSH cDNA libraries (Table 1, libraries E1, E2, M1, M2, S3, S4 and Z1) were generated using standard high throughput sequencing by GATC Biotech AG, Germany. The DNA templates were subjected to single pass automated sequencing using the ABI3730 (Perkin Elmer, Foster City, CA, USA). The sequences were then subjected to an automated procedure to verify cleanse, store and analyze sequences as previously described [16]. The automated analyses allowed the identification of potential unigenes (contigs plus singletons) through simultaneous cluster analysis. Finally, to assign putative functions to the ESTs, BLASTX http://www.ncbi.nlm.nih.gov/BLAST/ was used to compare sequences with the GenBank non-redundant protein sequence database as previously described [28]. The ESTs were manually assigned to functional categories based on a previous catalogue system [22]. In addition, Gene Ontology (GO, http://geneontology.org/)-based annotation was performed using Blast2GO to assign GO molecular function, biological process and cellular component terms [29]. The sequences were analyzed using BLASTX against a GO-based plant uniprot database with an E-value cutoff of 10^-10^. To identify SSRs within the oil palm EST collection, the online SSR Analysis Tool (SAT; http://sat.cirad.fr/sat) with the default parameters for the SSRIT program was used [30]. The complete list of *Elaeis guineensis *EST-SSR loci with their EST GenBank accession numbers, derived primer pairs, melting temperatures and predicted PCR product sizes are included in Additional file 1: Table S1. An annealing temperature of 52°C and an MgCl_2 _concentration of 0.6 mM was used for the PCR reactions performed as described previously [9]. The ESTs from the libraries E1, E2 and Z1 were submitted to GenBank and were assigned the accession numbers JK668500-JK669437, JK669438-JK669618 and JK668122-JK668499 respectively.

## Abbreviations

EST: Expressed Sequence Tag; GO: Gene Ontology; ORF: Open Reading Frame; SSR: Simple Sequence Repeat; SSH: Suppression Subtractive Hybridization; TF: Transcription Factors; UTR: Untranslated Region.

## Authors' contributions

TJT and WK compiled and annotated the ESTs, and performed the SSR search. FM and WK designed and validated primer pairs in flanking sequences of EST-SSRs. WK, DS and NB tested the EST-SSR primer sequences to detect polymorphisms in elite material. JWT and FM participated in the cDNA and SSH library constructions. TJT wrote the manuscript and with ST participated in the conception and coordination of the study. All the authors read and approved the final manuscript.

## Supplementary Material

Additional file 1 **Table S1**. List of 289 *Elaeis guineensis *EST-SSR loci with their EST GenBank accession numbers, derived primer pairs, melting temperatures and predicted PCR product sizes. Click here for file
