# Supplementary material for: SSR markers in transcripts of genes linked to post-transcriptional and transcriptional regulatory functions during vegetative and reproductive development of Elaeis guineensis
Source: BMC Plant Biol. 2012 Jan 3;12:1. doi: 10.1186/1471-2229-12-1 (PMC3282652; doi:10.1186/1471-2229-12-1)
Supplement: Additional file 1 — Table S1. List of 289 Elaeis guineensis EST-SSR loci with their EST GenBank accession numbers, derived primer pairs, melting temperatures and predicted PCR product sizes. [file 1471-2229-12-1-S1.DOC]

**Supplementary Table I** List of 289 *Elaeis guineensis* EST-SSR loci with their EST GenBank accession numbers, derived primer pairs and predicted PCR product sizes

| GenBank N° | SSR Locus | SSR motif | 5'-3' Forward Primer^1^ | 5'-3' Reverse Primer | Product^2,3^ |
| --- | --- | --- | --- | --- | --- |
|  |  |  |  |  | size (pb) |
| CN599381 | mEgEST001 | (acc)5 | ATGGCTACCTCCTCCCTA | GATGGAGTTGAGGTCGTACT | 191 |
| CN599385 | mEgEST002 | (tc)6 | CCACAGGTCACAACAATCCA | TCATCAGCAGCAACTTGGAC | 167 |
| CN599409 | mEgEST003 | (cgc)5 | CACGATGTTCTCGCAGGT | GCTTCCGTGAATTTCCATAG | 200 |
| CN599614 | mEgEST004 | (at)10 | GAGTGGCACCTGGAAATC | CCAGGAAGTTTGTGGAGAG | 215 |
| CN599645 | mEgEST005 | (tgg)6 | ATTTCTTCTGGGCCATTGTG | TTTAGCCTCCACAGCAGGAT | 247 |
| CN599688 | mEgEST006 | (agca)3 | CGATCCCTTTGCTTCAAGTC | GCCCAAGGCTAATGCTATTG | 184 |
| CN599755 | mEgEST007 | (gttagg)3 | GCATCCCTTTTCTCTTCCTA | ACATCCTCACAGGGTCAAT | 189 |
| CN599840 | mEgEST008 | (ct)17 | GGCACATAATTCCCAACCAC | CCCCAGTTTGCTGAATAGGA | 241 |
| CN599912 | mEgEST009 | (cttt)4 | ATCCATGGCTCGCTTCCTA | GGCGCAAGTCCTTCTCTATG | 241 |
| CN599963 | mEgEST010 | (ttttgg)3 | AAGCGCAAGGAGATCGTAGA | GAGGATAGAACCCAACAGACTGA | 173 |
| CN599997 | mEgEST011 | (acc)5 | GCACATCCTACGGTTGGTTT | ATGGAACGGCAAGAGAACAC | 191 |
| CN600082 | mEgEST012 | (aaat)3 | CAACAACGAAGGCCGATAAC | TCCGTTCAAGAGGAAGCACT | 224 |
| CN600084 | mEgEST013 | (ag)16 | AAGGGAAGCTCGGAAATGTT | TCTTGGTGTAGGTGGTGTCG | 223 |
| CN600053 | mEgEST014 | (ct)6 | TGCTAGAAGTCTCCCCACT | CCGATAGACAACGAGAACAT | 201 |
| CN600203 | mEgEST015 | (gga)5 | TAAGGAAAATGGGGTATCG | CTCTCCCACCCTTCAGAT | 168 |
| CN600268 | mEgEST016 | (gga)5 | CACACCTTCCCTCTCTCTCG | CATAATCCGTGGGGAAAGTG | 206 |
| CN600307 | mEgEST017 | (aaga)3 | AATGGCAGTCTCAACCAAGG | GGAACACACCTGCACTTGAA | 197 |
| CN600371 | mEgEST018 | (gaac)3 | GTGGAATCAGCTGCCAAATC | CAATCAGGCTCCAGTTCCTC | 216 |
| CN600400 | mEgEST019 | (ggga)3 | TCAGAGCTCAGACGCAGAGA | CAACGAGGAGGGAGAGAAGA | 193 |
| CN600508 | mEgEST020 | (ggaa)3(ttaggg)3 | CTACCCTGGCATCCTCTCAA | CTCCTACCCCTCCTCCTTTC | 163 |
| CN600573 | mEgEST021 | (aaga)3 | TACGACTTCCCCTCCAAA | GCGAGTCTCCTCGTAGATG | 204 |
| JK668571 | mEgEST023 | (gat)7 | AGCCACTGAAGGAGGATATT | CTGAGATGTGTCTCCCTGAT | 203 |
| JK668591 | mEgEST024 | (ga)6 | TTCCCCTGCACTTTGCTTCT | ACGCGACCCCTACGATCA | 177 |
| JK668629 | mEgEST025 | (gat)5 | TGACGGACCTGCAAAGAAAT | AGCTGCAGCAAACACAGATG | 219 |
| JK668665 | mEgEST026 | (ga)9 | CGACGGAAACCATATTCGAT | GGGCCAACAATCATAAGCAG | 226 |
| JK668687 | mEgEST027 | (agac)4 | CGCATTAGCTCCACCAGAAT | GCGATCTGAATCCTTTGCTT | 167 |
| JK668742 | mEgEST028 | (ga)7 | GGCGGATACAGATTGGGTTA | AGGAAGGATCTGGCTGCATA | 245 |
| JK668766 | mEgEST029 | (tgc)5 | GCTAAACGATCCGAGAGTAA | TTAGGCATCTTGAGTCCATC | 196 |
| JK668790 | mEgEST030 | (tttg)3 | GAATCACAGCGACTGATGGA | AACCTGGTTTGGAGCAGAAA | 180 |
| JK668803 | mEgEST031 | (aaaat)4 | GGGATCGTCCTCTGAAAT | GGAGAAACCGAGAAGGAC | 197 |
| JK668830 | mEgEST032 | (gagc)4 | CAGTCGCACACGTTTTTGTT | TATTCATGGCTCCGTCCCTA | 226 |
| JK669024 | mEgEST033 | (gaa)5 | TCCATGTTGATCCGATGTTG | AAAGAATGCATCCCAAGTCG | 173 |
| JK669075 | mEgEST034 | (ga)16 | GCCGAAGAGAATCGATCAAG | CCTCTTTCCTTGGATCTTGC | 249 |
| JK669080 | mEgEST035 | (ct)6(gtgcgc)3 | GGGAAGAGCGACCCAAAT | GCGGTGCTTAGATTGAGGAG | 221 |
| JK669086 | mEgEST036 | (tcaa)3 | GGTACTAGTTTGCGCCAAGG | GGGAAGGGATGGTAGTGGTT | 222 |
| JK669144 | mEgEST037 | (attg)3 | TGACCATATCCAAGCGACAA | ATTGAGGTCACGAGGAGCAG | 204 |
| JK669188 | mEgEST038 | (ag)6 | AGCCCTGGAGAAAGTTCCAT | GGGGAGTGGAGATCCTAAGC | 201 |
| JK669192 | mEgEST039 | (tgcg)4 | TTTGAGGAAGGTGGGATGAG | CGTCCACCATGTCATACGTC | 241 |
| JK669223 | mEgEST040 | (cgaa)3 | TGTGACCCATACTGCTTCTGTT | GGAAGCATCTGCAAACAGTG | 232 |
| JK669305 | mEgEST041 | (ttgat)3 | CCCTTCTGCCACCAGATTAC | GCCTTGTTGAGGGATCTTGA | 170 |
| JK669327 | mEgEST042 | (gag)6 | CGTCTCCTTTGAGCTTCGAG | AAACGTTCCAAGCCAAACAC | 238 |
| JK669433 | mEgEST043 | (ggga)3 | GACGCAAAAGCCTCAAAGAC | GCGAAGATGAAGAGCATTCC | 206 |
| JK669500 | mEgEST044 | (aga)5 | CCTGCCACCATCTCTAGCTC | GGGGAAATTAGCCTTCCAAA | 223 |
| JK669501 | mEgEST045 | (agaga)3 | GATCCCCGAGAGATCGAGAC | TCTCCATCCTCCATCCCATA | 174 |
| JK669530 | mEgEST046 | (ct)16 | CATTCGCGCACTAGAAAC | CCGATCTATTCCAGAAGCTA | 217 |
| JK669557 | mEgEST047 | (gaaa)13 | CCTCCCAAATGAAAATTGCT | TGAAGGGGAAGTTGCAGAAG | 239 |
| CN600757 | mEgEST048 | (ag)9 | GTTCGAGGTGACTGCTGGAT | AGCCATGCCCGCTTATTT | 195 |
| CN600784 | mEgEST049 | (ccg)6 | TCGCTCTCCTCTCGATTGTT | GTACTGGTCGGCCCAGAATA | 224 |
| CN600805 | mEgEST050 | (ct)18 | GCAGGTCACCGAACCCTAAT | TGAGGACAAAAAGCCAAAGG | 161 |
| CN600809 | mEgEST051 | (tc)7 | CTGCATCAGCGCAAAATA | CATCTCCAAGAAATCCAGGT | 216 |
| CN600830 | mEgEST052 | (cgtcgc)3 | CGTCTTTCGAGCTGCTTCAG | TTCTGAGCTTTCTTGGACTCG | 241 |
| CN600893 | mEgEST053 | (tg)7(gt)8 | TTCCCCTTGAACTCTGTGTG | TCCCCAACGGTAACACATTT | 153 |
| CN601180 | mEgEST054 | (ct)9 | ACCCATCAAATCCTAGACCT | AGTATCGCAGAACACCAGAT | 206 |
| CN601255 | mEgEST055 | (tc)7 | GCATACACATCAGCAGATCAAAA | GCCCATGGTTTCCACTTCTA | 150 |
| CN601280 | mEgEST056 | (ct)7 | TCGCCTTTCCATTTGTCG | GTACTTGAGCTTCGGCACCT | 150 |
| CN601295 | mEgEST057 | (ataa)3 | CACCTCATAAAAGGCAGTGT | CGAGTCAATCTCCTCAGTTT | 194 |
| CN601340 | mEgEST058 | (cag)7(tttttc)3 | CCCTTTTCCCCTCGACTT | CCCGGGTGGTGATAAAGG | 240 |
| CN601359 | mEgEST059 | (ta)6(ag)7 | GCCATCTAACACTCTTCCAC | CTAACAGTGGCTCTGACGAT | 230 |
| CN601405 | mEgEST060 | (ct)8 | CAGCTCCCGGTCTTCTCC | ATCTGCAGCCGAAGAGAGAG | 162 |
| CN601439 | mEgEST061 | (cag)6 | CGCTTCGATTGAGAGATGGT | CCGTTCTGGCTCAAACTGAT | 215 |
| CN601451 | mEgEST062 | (ccg)5 | TGTTGTATGTGGTAGCGTTG | TGTAGATGGAGGAGGTGATG | 215 |
| CN601537 | mEgEST063 | (gaa)6 | TGGTTCTGCAGGTGACTCAG | CCATCCTCCTGAGCTTCATT | 170 |
| CN601623 | mEgEST064 | (ccgcgg)3 | CCTAATTCTTTCAGCCACAG | GACCCTCCTTCACCAAAC | 204 |
| JK669619 | mEgEST065 | (caaaaa)3 | CGCCTCTCTAAGAGGAGCAA | ATCAATGGGGTTAGCTGCTG | 161 |
| JK669620 | mEgEST066 | (ttggc)5 | ACAACTCCTCTCCCGTTCTG | CGTCCACGTGAGAGAAAACA | 175 |
| GT119243 | mEgEST067 | (tct)6 | CCAAATGGACATCTCCTCGT | AATCTCGACAGGAACGAAGC | 191 |
| GT119345 | mEgEST068 | (tca)5 | TCTTCTTGGGAGTAGCTTCA | AAGGCTGATAAACAGAATGC | 192 |
| GT119445 | mEgEST069 | (ttc)8(aaag)3 | ATCTTCCCCTCCAGATGTAT | CATTACCCTGATCCTTCTCA | 253 |
| GT119554 | mEgEST070 | (tgc)5 | TCCTCTTCAACGATGGCTTT | GGAGCCCCAGAACTCTTCTT | 176 |
| GT119614 | mEgEST071 | (tcc)6 | CGAGGCATGGAGTGTCCTAT | AACGTATTGGGCTTTGATGG | 251 |
| GT119614 | mEgEST072 | (atcctc)3 | CGTATCCAGGCTCTCCGTAA | GAAGCCAAAGCTGCAAAAAG | 203 |
| GT119730 | mEgEST073 | (tc)6 | CACAAGAAAGACCCCTTCCA | GGCTCTTCTTTCGTTCCAAAC | 220 |
| GT119741 | mEgEST074 | (cag)5 | GAAGTGTTGCATCTGCTGGA | CACACCACAAAACCATCTGC | 243 |
| JK669621 | mEgEST075 | (at)6 | CGCGGGAATCTTGTTAATATGT | GTGGCCTCACCTTTACATGG | 238 |
| GT119777 | mEgEST076 | (tc)6 | GAAATCTGAACTTCCATCTCA | AGTTTGCTTAACAGCTTGATG | 241 |
| GT119797 | mEgEST077 | (ccga)3 | CTCATGGGCAGCCTCATATT | AAGCGAGGAAAAGAGGGAAG | 235 |
| GT119801 | mEgEST078 | (gtg)5 | GACTTGTATTGCACGATGG | GTCATCTGCTCACTCTCGTT | 216 |
| JK669622 | mEgEST079 | (tct)5 | GCATTGGTTCCCTTAGCAGA | CAGCAGGTTGATCTTGGTGA | 196 |
| GT119857 | mEgEST080 | (tc)10 | CCAGTGCCATTGTCACAGAC | GTCATCCCTTCGGCCTTG | 191 |
| JK669630 | mEgEST081 | (ttttc)3 | CTCCCCTCCAATACCCAAAT | GGAACCGTCGTCGCTAGTTA | 180 |
| GT119989 | mEgEST082 | (ggaag)3 | GCTTCGGCTACATGATCTCC | TCCAGGGGTTCTTTTCCTTT | 215 |
| GT120021 | mEgEST083 | (ct)8 | CTTCCCCAACGCACACTTAC | TCCCTGCAAAAGGACTCAAC | 230 |
| GT120031 | mEgEST084 | (catt)3 | GATCGGGGTTTCCCAAATAC | TCGGTGAAGAAGCTACATGG | 236 |
| GT120080 | mEgEST085 | (ga)11 | GCCGAGAGCTTACAAAGTGC | GTGATGGGCTGATGGCTTAT | 162 |
| GT120157 | mEgEST086 | (ctc)5 | TCTGTGCCATGCTGAAACTC | TCCTTCTCCGAGTGATCGTT | 216 |
| GT120245 | mEgEST087 | (ccttct)3 | TGGTCTCGTTGCAGCAGTAT | TCTCCTCTCCGTGGTTGACT | 248 |
| GT120315 | mEgEST088 | (gga)5 | GGCATACATTCACCCCACTT | TTCTATGCCGGCACTCTCTT | 180 |
| GT120438 | mEgEST089 | (ct)9 | AGCTCGTATGCTTTCTTGAG | TAGCTTCTTCCACCATCCT | 178 |
| GT120444 | mEgEST090 | (tc)7 | GAGGAAACAAACATGATGTGG | TAGGGGCTGTGGCAGATAAA | 251 |
| GT120479 | mEgEST091 | (ctcc)3 | CAAGCCACCAATTGTCAAGA | ATGCAAAAGCCTCAAAGACG | 184 |
| GT120517 | mEgEST092 | (ctttt)3 | ATAAGAGCGCTGGCAAAAGA | GGGGCTCTCTCCCTTCTCTA | 183 |
| GT120648 | mEgEST093 | (ct)9 | GCCTTTCAAGTGTCTTCATC | GATTCTCTTCCAGCGATCT | 256 |
| GT120666 | mEgEST094 | (aaag)3 | AAGGTGAAGAGGACCCCAC | GCCACCAAGGGACTTTCTTT | 181 |
| GT120668 | mEgEST095 | (tgaga)3 | GCTTTTCGAAGGTCCTGTTG | CGAGAAAGGAACTTGTGGTTG | 189 |
| GT120710 | mEgEST096 | (atgg)3 | CAAATCTCATGGGCAAAACC | TAGAGCACCATCCACACTGC | 239 |
| GH635931 | mEgEST097 | (ttgg)3 | TTGTGGCCACCTAATGACAA | AGGTTTCACAGTGGGTCTGG | 244 |
| GH635945 | mEgEST098 | (ttc)5 | CTTATCCACCGCTCCATTGT | AACGGTGGGATGAAGTATGG | 239 |
| GH635946 | mEgEST099 | (aagaaa)3 | AGGCAGCAACGTTCAGATGT | TGGTTGGGCATCTCTTTTTC | 223 |
| GH635970 | mEgEST100 | (tct)11 | GATGTTGACGAACTCAAGGT | GTCAGAGCAACGAAGAAAAT | 244 |
| GH636078 | mEgEST101 | (tcga)4 | AACGCCTCCTTCTTCGTTTT | GGCTTTCCGAAAATCCTACC | 154 |
| GH636104 | mEgEST102 | (aagg)3 | CCTGAGGTTTGATTGGGAGA | CGATTCCTTGGTAGTGACGA | 247 |
| GH636120 | mEgEST103 | (ttctt)4 | AAACAATCCTCCGAAGCAGA | TTGGTTTCCCAGAGTTTTCC | 239 |
| GH636141 | mEgEST104 | (ga)9 | GGGGAAGTAGTCTTTGCAT | CAAGGAGGATGTCGAGTTT | 194 |
| GH636211 | mEgEST105 | (ct)6 | GAACCGCGTCTCTTTCAT | GGTACGTCATGTAGTCAGTGC | 213 |
| GH636222 | mEgEST106 | (tggc)3 | TGTCCACGAATCTGCACTGT | ATGAGACAGCCAAGGCATTT | 184 |
| GH636326 | mEgEST107 | (ctccct)3 | GTCATAAGCCGACCAAGAGC | CTCCAAACCCTTCTCTGCTG | 173 |
| GH636390 | mEgEST108 | (acc)6 | TGGTTTTGCTCCTGATTTCC | GGCAGTAGATCACCCCTTCA | 173 |
| GH636397 | mEgEST109 | (ctt)5 | GAAAATGGGCGGTGGAAT | CAAGTTCTTCAACGGTGCTTG | 193 |
| GH636466 | mEgEST110 | (ggga)3 | AGGAGCACCACCACTCAAAC | ACTACAGGGCACCACACAGA | 232 |
| GH636479 | mEgEST111 | (aacc)3 | CAAGGAGAACATGACGCAAA | ACTGATTAATGCCGGTTTGG | 180 |
| GH636491 | mEgEST112 | (cct)7 | TGGAGGTCCAAACTCGAATC | CCGACCCTCTTCCAAGAAAT | 173 |
| GH636515 | mEgEST113 | (gag)5 | CATTACCGGTCAAGGTTTGC | GACGCTTGGGTAGCTGAGAC | 168 |
| GH636588 | mEgEST114 | (tcc)5 | CCTCTCCGAGAAAGTCATCG | TCCAAACCTCCCCAAGAAAT | 163 |
| GH636604 | mEgEST115 | (ga)9 | ACCCTTCTTCCGCCTACTGT | ACAATGGAGATCCCAGCAAG | 173 |
| GH636623 | mEgEST116 | (cctc)4 | TATCCGCGATCCGGTAGTAG | GCCGCCGAACTCTACAATTA | 241 |
| GH636728 | mEgEST117 | (ag)12 | TGATAGGATCCGCTTCCTTG | TCGGCTCTACTGGAGTCTGG | 226 |
| GH636730 | mEgEST118 | (aag)5 | AGGAAGGCAAGGTGGAAGAT | ATGCTCCTCCCAGTCATTTG | 196 |
| GH636774 | mEgEST119 | (agc)6 | GAGAGAGCTCGGAGGAGTCA | CTGTCTGGGGACCATCTTTG | 161 |
| GH636880 | mEgEST120 | (cttc)3 | CGCACTTCTTCCTTTTCCAG | AACGGGACAGTGAGGCTTTA | 195 |
| GH636899 | mEgEST121 | (ctc)5 | ACTCGTTGGGCACGAAATAC | ATCGAGACGGCACTATGACC | 188 |
| GH636902 | mEgEST122 | (atac)3(tatatg)3 | TGTGATACTGCGTCCGAGAG | TGGCTCTGGAAGCCTAAAAA | 211 |
| GH636952 | mEgEST123 | (tgc)5 | AGGTTGAGGCTGTCATTGCT | CTGTTGCCATTCTACCACCA | 160 |
| GH637030 | mEgEST124 | (ga)9 | CCTGAAGCTTCTCGATGGAC | CCTTGTCGTCTCCACTCCTC | 224 |
| GH637036 | mEgEST125 | (ttct)3 | AAGCATCCCACTTGGCTCTA | GCCCAAAAAGATCTCAAGCA | 232 |
| GH637071 | mEgEST126 | (tgg)6 | GCATGGGTAAGGCTCCTAAA | CGACAAAGCAAAGTGCTTCA | 186 |
| GH637088 | mEgEST127 | (ttta)3 | CCAAGAAGTTGATAGCGAGA | ATGAAAGATCCCATTGAAGC | 212 |
| GH637106 | mEgEST128 | (ct)14(ta)8 | GCCCCTATGATTCTCACCAA | GCCTGTCCTCTAATCGAGCTT | 245 |
| GH637153 | mEgEST129 | (tc)7 | CAACCCCCATTTTCATCAAG | AAGGAACATGTGCAAGAGCA | 187 |
| GH637195 | mEgEST130 | (ct)6 | CAGCTTCACTACCTTTCTGG | GGCTCCTCTGAAACCATT | 193 |
| GH637350 | mEgEST131 | (cata)3 | AGCTGGTGGTGGCTATCAAG | AACATGCAATGAAGGGGTTC | 171 |
| GH637470 | mEgEST132 | (ctcc)3 | AAATTAGCAACACTAGCACTCA | CATCTCATTTGTTGTGCATC | 270 |
| GH637534 | mEgEST133 | (ag)6(ag)7 | TTTCTCCTCATTTCTTCTTTT | GCTAACAAACTTCCTCCAG | 285 |
| GH637545 | mEgEST134 | (tga)8 | GGTGCTGGTGTGAAAATTGA | CATCATCCCATGGCTTTACA | 252 |
| GH637594 | mEgEST135 | (aaat)3(aaacaa)3 | CACTGGTATCAACGCAGAC | TAAGTGACCGGATGAAGTTT | 176 |
| GH637606 | mEgEST136 | (ctcc)3 | GGAGGAGATGGTACAGACAA | AGTTGAAGGGTTCGAGATG | 199 |
| GH637609 | mEgEST137 | (agc)5 | TGGAGCTGGATGAGTGTGAG | CCAAAAGGTGCACTTACTGCT | 158 |
| GH637610 | mEgEST138 | (tc)8 | TTCCCATGGGTCGAACTTAC | CGGGGAAACACCCTACCA | 217 |
| GH637669 | mEgEST139 | (ttccat)3 | CAAGGTCTCTTTGCCGTTTC | AACTTGCGCCAAGGAAGTTA | 211 |
| GH637746 | mEgEST140 | (gcaa)3 | CCTTGTGTGGTGGTGACATC | CCATGAGGGCTGTTTGGTAT | 234 |
| CN601771 | mEgEST141 | (tatg)5 | TGAGAGCCATAGAGATAGCC | TAGACCCGACCATTAAACC | 149 |
| CN601720 | mEgEST142 | (gag)5 | ATAACACCGAGGCAACCAAG | TCGACTCTTCGGTAGTGCTG | 192 |
| CN601727 | mEgEST143 | (gt)8 | GAGGAGCAGTCGATATGTTT | TTACATCATAAGCCTAAATTATTACA | 143 |
| CN601741 | mEgEST144 | (gcg)5 | GGGGAGCTGCTTCTTTCTCT | GACCCCAGCCATGATGTACT | 181 |
| CN601754 | mEgEST145 | (cgtc)3 | TCCATGCAGTACCCCTTC | GGGCTGAAGAGGAGAAAGA | 260 |
| JK668145 | mEgEST146 | (tttc)3(ttttc)3 | CTGCTATTGATGGAAGAAGC | TATCGAACCTCTCAAGCAAC | 203 |
| JK668249 | mEgEST147 | (ctt)5 | ATTGCCAACTGCTGAGCTTT | GGGTAGAGGCAGAGGAAAGG | 234 |
| JK668361 | mEgEST148 | (cttc)3 | TCCTTGTGGATGCCTTCTTC | TGTGAGTGGTTGATGGAGGA | 162 |
| JK668363 | mEgEST149 | (aata)3 | TTCTCGTGGTCCAGAATCAA | GGTTTTGTTGCGGAATCAGT | 209 |
| JK668407 | mEgEST150 | (tttg)3 | GGACATTCAGCTGGCTAGGA | TCCATTTTTCCCCATACCAA | 248 |
| JK669623 | mEgEST151 | (ga)15 | AAACTCCCTATCATCCTTCG | TACGTGGGTCACAACAAAA | 196 |
| CN601607 | mEgEST152 | (cgat)3 | AACAACCGTGATGTCGTTGA | ACAGGTGCTCAGCGAAAAAT | 152 |
| CN600583 | mEgEST153 | (gcag)3(ggcgcc)3 | AACTCCACGCTTAGCCATGT | AGTTCTTGTCGGGCACCTT | 210 |
| JK669618 | mEgEST154 | (ct)6 | AACTTCCCCTCCTTCCATGT | TGGTGAGAACTCCAACGAAA | 185 |
| CN599671 | mEgEST155 | (gcc)5(cgg)5 | ACACCAATGCTGCTGTTGCT | AACTGCCATTGGGAATCATC | 185 |
| CN599923 | mEgEST156 | (aaac)4 | ACATCCCATTTGACTTGTTC | CGTAGAAATCCATTCCGTTA | 162 |
| CN600516 | mEgEST157 | (tatg)3 | ACCGCAGAGCGATATTGTTT | TGACACAAGAAAAGTTCACCTCA | 235 |
| JK668797 | mEgEST158 | (ga)13 | ACCTACTGGTTGGCAGGTAT | ATAGTTCAGAAAGGGCTGGT | 186 |
| GT119121 | mEgEST159 | (cct)5 | ACTCTTCTACTGCTCCACCA | GCAAACTCCCAACTTTTCTA | 195 |
| GT119704 | mEgEST160 | (gatc)4 | AGACGAGATCCGACGAGAAA | GATCCATCGAGAAGGTTGGA | 201 |
| CN601122 | mEgEST161 | (tc)8 | AGATGGCCGAAGACAAGGTA | CTCCGAAGGACAAGAAGACG | 174 |
| JK669624 | mEgEST162 | (caaa)3 | AGCACACTGCAGCGATTTTA | ACCCATCCAAAAAGCAAAGA | 216 |
| CN599851 | mEgEST163 | (tgct)3 | AGGAAAGCAGTTGTTCTGTGG | CCTCCCAGCATCCTACTCAA | 234 |
| GT119420 | mEgEST164 | (gcagta)3 | AGGAGATGTTGGAGCGAGAA | CAGAATTCGGGTCTTCGTGT | 170 |
| CN600338 | mEgEST165 | (atcc)3 | AGGGTGGAGACCCAATTGTT | ACCATGGTTGCCGAGAAAT | 228 |
| CN600270 | mEgEST166 | (agc)6(cca)5 | AGTACGCCATATCCCCATCA | CCAGCTCCTCATCCTCACTC | 185 |
| CN601329 | mEgEST167 | (tc)7(gagc)3 | ATAGCCGTAAAGCCAAACGA | TCAAAGCCACCAATCACTCA | 216 |
| GH637298 | mEgEST168 | (gtct)3 | ATCAAGCCTAGCGCAGAATG | GATCCCCCACTTCTTGTGAA | 209 |
| JK669588 | mEgEST169 | (aaat)4 | ATCCGTTGCACAAAACCTTC | CATGTCGGCGAAATAGGATT | 200 |
| GH636772 | mEgEST170 | (ctt)6 | ATCCTCTGGGTTGAGGTAGT | GATGAGACAGGAGGGAAAA | 217 |
| GH636801 | mEgEST171 | (tgg)7 | ATCTCCCGAATCCCCTAATG | TTGGATTGGAGTCTGCCTTC | 182 |
| JK669613 | mEgEST172 | (tcct)3 | ATCTCTCGCGCTGTCTCTTC | GAGATGGCCATAAAGCAAGC | 239 |
| GH637214 | mEgEST173 | (cttgt)3 | ATCTTGCTTTCCCCTGACCT | GCCCCGGGAAATTTATTAAG | 208 |
| GH636624 | mEgEST174 | (aagc)3 | ATGCGATGCAATAAGCACTG | TGTCATGAAGGCTCTGATGG | 228 |
| JK668301 | mEgEST175 | (ct)18(tg)6 | ATGGTAGGCAAAAGGGTGTC | CAGTGGTCTCCCCTCTCTTG | 183 |
| GH636732 | mEgEST176 | (gaag)3 | ATGGTTCACCCAACCTTCAG | TCCCACTCTGGGTTTGAAGT | 180 |
| JK669008 | mEgEST177 | (catc)3 | ATTCTTGACGCCTGCACCT | GGACCCTAGAACCCGAAAAG | 202 |
| GH637405 | mEgEST178 | (attaa)3 | ATTGCTAATGCCGGACAACT | TGTTCAGTGCCTTGCACATT | 155 |
| GT119971 | mEgEST179 | (tca)10 | ATTGCTTGCCTGCCTTAGAA | AGGATGGCCACAAGAACAAC | 217 |
| JK668723 | mEgEST180 | (tgcc)3 | ATTGGGTGCAGCCTCTGTAA | AAGGGCTTTCCAAGATCCAT | 208 |
| GT119149 | mEgEST181 | (taaa)3 | CAAGGCTTCAACACGATTCA | CCCAGGCTTGATATCTCTGC | 230 |
| GH636140 | mEgEST182 | (tgcc)3 | CAATAATGCACGTCCTGGTG | GTTGACATGCGCCTCAAGTA | 185 |
| GT120288 | mEgEST183 | (ct)7(tc)6 | CACCGCAAAGTGGAAGACTC | ATTGGAATTGGGATCAGTCG | 214 |
| GH636826 | mEgEST184 | (ag)9(gagg)3 | CAGAAATCCTGCCAAGGAAA | CCCTTCGATCTCACTGCTCT | 222 |
| CN601001 | mEgEST185 | (cag)5 | CAGCAGTGATTGGAGCCATA | TGAAGTCATTGGCATGCTCT | 167 |
| JK668810 | mEgEST186 | (ct)14(cctc)3 | CAGGCACTCCTTTACCAG | CCTCTTCGTTTCAGTCCTC | 211 |
| GH636001 | mEgEST187 | (at)7 | CATGGAACAGTCTAGCCAAA | AAATGGGTCAGGTTATGGTC | 192 |
| CN600653 | mEgEST188 | (acc)6 | CATGGAGACCGTGGAAAC | CCTTAGAGCGAAATCCAAAG | 132 |
| CN601454 | mEgEST189 | (tctcc)3 | CATTCTGATTTCTTCCCCAAT | GGAAAAGTAAGGCGAAGACG | 232 |
| CN601056 | mEgEST190 | (ccg)8 | CCAATTTCCGATTCCAAATG | GCCACAGAATCCTCTTCAGC | 153 |
| GT119292 | mEgEST191 | (ac)7(agac)3 | CCAGTTAGGACTGACCGATA | CTCCTTGCTCCTCTTTGTC | 201 |
| CN600785 | mEgEST192 | (ct)10 | CCCACCCTCATTTCTCTATT | CTCAACAAAATCGGCTCTAC | 130 |
| GT119541 | mEgEST193 | (ag)14 | CCCGATACACCTTTACTGTC | CATGAGGTCTCCATTTTCTC | 225 |
| JK669625 | mEgEST194 | (ct)7 | CCGACATATCCTCGAACTAA | GTTCCCATCTCCGTTCTC | 224 |
| GH636514 | mEgEST195 | (tccc)3 | CCGATTCCTCCTTTCTTTCC | CCTGTTCAACTCCTGAGATTCC | 182 |
| CN601492 | mEgEST196 | (cct)9 | CCTCCAAACACCCCAACTT | GCTCGCACTCCCAGAAGA | 178 |
| CN600638 | mEgEST197 | (ctc)5 | CCTCCACCCTTTCTAGTATTTCC | GGCTCTCTTCCTGTGTCTCC | 149 |
| JK668513 | mEgEST198 | (tc)8 | CCTCCTCCCCTCCTTACCTA | GCCGATATTTTCTCCGATCA | 193 |
| GH637485 | mEgEST199 | (tc)7 | CCTCTTCCTGTCTTCCACCA | CAGGTTGGAAGGAGATGGTT | 250 |
| CN601599 | mEgEST200 | (tttc)3 | CGAACTGCAGGTCCACCTAT | TGGAATCCGAAACAAAACG | 239 |
| CN601087 | mEgEST201 | (ct)10 | CGCCTTCATAACTCTCAACT | AGCGAGCACCAGAAGATA | 221 |
| GT119833 | mEgEST202 | (ct)6 | CGGCTTTGTAGGGCAACTTA | AGCCCATGAAAAGGACTCAA | 221 |
| JK668968 | mEgEST203 | (ttct)3 | CGGGGAGATTTTTGAAGAAC | AGGATAGCGGCCTTACCATT | 191 |
| JK668635 | mEgEST204 | (gatc)5 | CGGGGCTACTGAGAAGAT | ACCTCTTCAGCATATCATGG | 191 |
| GH637615 | mEgEST205 | (atta)3 | CGTGCAACATTATTGCCTAAC | CCTGTGTCCATGGGGACTAT | 231 |
| GT120490 | mEgEST206 | (cag)6 | CTCAGACCCAAAGCGAAAAC | GGAGTGGAAACGAAGATGGA | 204 |
| JK669486 | mEgEST207 | (ttga)3 | CTCCAATGTCCGCGACTAAT | CAGACGGGAAACCAAACAGT | 193 |
| CN599632 | mEgEST208 | (ggagag)4 | CTGCAGTTTGGTTGGGTGTA | AAACATGCTTTCAGGGCATT | 174 |
| GH636685 | mEgEST209 | (ataat)3 | CTGCTGCTTCTGCTGCTATG | CAAAGGAGCATTCTTCACGA | 240 |
| GH637671 | mEgEST210 | (acaa)3 | CTGTGCAAAGCACTTGGAAA | CGAGCTCTTTCTTCATGCTG | 177 |
| GH637238 | mEgEST211 | (aatc)3 | CTGTTGCAATGGAGGGTTTT | TCAAAAGAAAACTGACCAGCAG | 155 |
| JK668278 | mEgEST212 | (ttctt)3 | CTTTTCTAGGGTGTTTCGTG | AGCAACAGAGAATACGATCC | 189 |
| CN599993 | mEgEST213 | (aga)6 | GAAATCATGGGCGAAACC | AACCCATTGCTCTCAAGGA | 244 |
| GH637479 | mEgEST214 | (ctt)5 | GAACTCTGCCGAGTGCCTTA | TAACCACATAACGCGATCCA | 188 |
| JK668234 | mEgEST215 | (aag)5 | GAATCGCTCGTATTTTAACG | CTTCCTCTACTCGCAACATC | 240 |
| GH637557 | mEgEST216 | (ctcctg)3 | GAATCTTGGTGCCTTAGATG | ACCACCTAGTAAACCTGCAA | 220 |
| JK669138 | mEgEST217 | (aggag)3 | GACGAGACAAAGCGAGAAGA | CCCCTGATTTCGATCCAG | 214 |
| JK669626 | mEgEST218 | (ct)6 | GAGCACGCAAGCACTATAA | ACACGCAAAGGAAGAGAAC | 187 |
| JK668725 | mEgEST219 | (aaat)3 | GAGGGACAGCGTGAAAACAT | GCGAAGGATGATGGAGAAAA | 184 |
| CN601450 | mEgEST220 | (ccca)3 | GATCAGTGCTGTAAGGAAGC | TCTCCAAGATGAATGTCTCC | 192 |
| GT120094 | mEgEST221 | (gggaa)4 | GATCCGCAGGAAAAAGAGAA | CCCTAATTCGTGTTCGAGGA | 177 |
| CN599974 | mEgEST222 | (tta)7 | GATTTCCGGCCTCTCACC | CTATTCCCCTTCCTCCCATC | 183 |
| CN600741 | mEgEST223 | (gag)8 | GCAAGGAGTTCCTCATCTGG | TGCAACGTCCATAATTTACCC | 214 |
| CN600706 | mEgEST224 | (cga)7 | GCACAAGAAGGGCAAGACTC | GCAGTCGGTTTCTCTTCGTC | 170 |
| JK668318 | mEgEST225 | (tcta)3(atct)3 | GCACGTTGCTTTCTACCACA | CCTTCCCCGGATGAATACTT | 218 |
| GH637255 | mEgEST226 | (ctttt)3 | GCATGGAAACTTGAAAGTGG | AAGGCAGAAAAGGGTGGAAT | 231 |
| CN599831 | mEgEST227 | (agg)9 | GCATTTCTCGATACCCGTTG | CCAAAGTACAGCTTGGTGTTTT | 189 |
| GT119869 | mEgEST228 | (tc)6 | GCCAAGAATCCAGGAACAGT | GGATTCTGGGTTTCGAGCAT | 172 |
| JK669029 | mEgEST229 | (ggat)3 | GCCACCTTTGGACTCCATTA | GTTCCTCCAGAGACCGGATT | 247 |
| GH637562 | mEgEST230 | (ccaa)3 | GCCCCCAACTTAAAAGGAAA | GGTACCCTTTTGCCATGGTT | 248 |
| JK668455 | mEgEST231 | (tc)13 | GCCGTCGCATCTACGAAA | TAAAGGACCATCACGGGAAA | 240 |
| GT119923 | mEgEST232 | (ttat)3 | GCCTTGAACTCTGACCTCCA | ATGTCATGATTTGGGGAAGG | 172 |
| JK669498 | mEgEST233 | (tct)6 | GCGTTGGTTCCTTCTCTCAG | GGACCCCAACAGCTCAAGTA | 153 |
| JK669629 | mEgEST234 | (ta)9 | GCTCCCCATGGATACTGCTA | GAGGCAAGAGGATCTGCAAG | 247 |
| CN599984 | mEgEST235 | (ttc)5(gagg)3 | GCTCTCGGGGACGTATTATG | GCAATGCTTGCTTGATCTTG | 184 |
| CN599594 | mEgEST236 | (tc)6 | GCTGAGGCAATGTTTGGTTA | TGGTTTCCAGCTTCCTTTCA | 208 |
| CN600733 | mEgEST237 | (tc)6 | GCTGCTTTGGTGACCTCTTT | GAGGAGAAAGGGATTGTTTGG | 192 |
| CN601507 | mEgEST238 | (tc)6 | GCTTTCCTCTTCGTCCTCT | CGATCCGGTCTTTCGTAT | 226 |
| GT119817 | mEgEST239 | (ct)6 | GGAACCAGAGAACATGGTTA | GGATCTGATACAAGCACGTC | 207 |
| CN601039 | mEgEST240 | (cctc)3 | GGAAGAAAAATCCGGAGGTC | GTGGATCCGAACCTCAGAAA | 175 |
| JK668230 | mEgEST241 | (atcg)3 | GGAGAACAATGATCGCCTGT | TTCGAGTGCATTTGAGCAAC | 213 |
| GH637525 | mEgEST242 | (tatt)3 | GGAGAGCAAGCAGAGTCCAA | CGAGGAGAAGAAATCGATGC | 167 |
| CN600112 | mEgEST243 | (atac)4 | GGAGGCAAGTTTGCTACCTG | TGGAATGGTGCAGAGAATGT | 182 |
| GH636337 | mEgEST244 | (ct)7 | GGAGGTTTTGTCTAGGGTTT | AAAGATGAGAAGAGCAGCAG | 209 |
| JK668815 | mEgEST245 | (at)6 | GGAGTCCAAACCTATTTGAG | ATTCGTCCTACTCCTTCCA | 204 |
| GT119293 | mEgEST246 | (atc)5 | GGATTGCTACCACAAAACTC | AGGGGTTATGTCAAGAGGAT | 192 |
| CN600168 | mEgEST247 | (aaag)3 | GGCACATTTGACGATACCAA | GAGGGATTGGAGGGGTAGAT | 238 |
| CN601501 | mEgEST248 | (ctc)5(ttttc)3 | GGCATCTCCTCTGTCTTCTT | CCTTGGTCCATCATAGAGG | 221 |
| CN600250 | mEgEST249 | (tcc)5 | GGCATCTGACTTCACCATCT | CTCCCCGTAGCTGAGGAT | 172 |
| JK668609 | mEgEST250 | (ga)7 | GGGGGATACTGACACAAGGA | GTCAGATGCCTCCTCGTCAT | 206 |
| JK669533 | mEgEST251 | (ct)14 | GGTCAAGCTTTGATGGAGGA | GAAGCAAATCCAAGCACACA | 164 |
| GH636292 | mEgEST252 | (tttc)3 | GGTGCTTATCGCAATGGTTT | TGCTCAGCAACCTGAATTTG | 178 |
| CN601779 | mEgEST253 | (gagg)5 | GGTGGCCTCTCTACTCTCCA | GAAGACATTTCACGCAGCAA | 238 |
| GT120202 | mEgEST254 | (atag)3 | GGTGTTGTTGTGGAGGATCA | CGACATGGGACAAACAACTG | 161 |
| JK668730 | mEgEST255 | (tc)6 | GGTTTTCCCTCTCATCTACC | CAGCATGACCTAGCAAAGAT | 209 |
| CN601488 | mEgEST256 | (agca)3 | GTCCCTTTGCTTCAAGTCCA | GCCCAAGGCTAATGCTATTG | 180 |
| GH636851 | mEgEST257 | (tc)7 | GTCGGGATCGTCGAAAACT | GATCCGTCCCCTTTCTTCAT | 204 |
| CN601335 | mEgEST258 | (gggtc)3 | GTGTTGGTGGAATGGAGCAG | GGACTGCAGATCGACAGGAT | 238 |
| GH636298 | mEgEST259 | (ct)8 | GTTCCCTCCCAAGGTTTCTT | TACCGATTCGGAAAGGACAA | 218 |
| JK668295 | mEgEST260 | (ga)10 | GTTCTGGACGGATTCAAACA | GCTTGGCACCTGGAAGTC | 113 |
| CN601436 | mEgEST261 | (ct)6 | GTTGGGAGAGGTTTCTGTTT | ACCTCCAAAGGGTAGTCCT | 169 |
| GT119794 | mEgEST262 | (acca)3 | GTTGTCGAAAGGGCTAGCAG | GCACAAGGAAGAAGCTTTCG | 196 |
| CN599384 | mEgEST263 | (tct)6 | TAAGAGCGCTGAGGAAATCG | GCTAGAGATTGAGGGTCTCGAA | 207 |
| GT120184 | mEgEST264 | (tttc)3 | TAATCGTCAAGGGCGATAGG | TTAGTCCACGGGATTTCAGG | 240 |
| JK669343 | mEgEST265 | (tc)7(ctcc)3 | TAGTCCACCATCGGTCTTCC | TACGGGATGGAATGATGTGA | 178 |
| JK669041 | mEgEST266 | (ct)11 | TATGATTTTGGTGCGGCTTA | CTGTTTTTGTGCGTGTATATGTG | 187 |
| GH637439 | mEgEST267 | (ttcgca)4 | TCACCACCTCCTTGAAAAGC | TAGAACCAATTGGGCAAAGC | 230 |
| JK669043 | mEgEST268 | (ct)6 | TCATCTTGCGATGATTGTCC | CGCTGAGACATGACACACAA | 217 |
| GH637053 | mEgEST269 | (ttc)5 | TCGATTTGTCGCTTCAGATG | CCGAGACGTCACTGGAAAAT | 180 |
| GT119141 | mEgEST270 | (tc)6 | TCGGATCGGTTCAGTCCTTA | TTCCTATTGCTCTCGGTGCT | 205 |
| GT120723 | mEgEST271 | (aatg)3 | TCGGTGAAGAAGCTACATGG | GATCGGGGTTTCCCAAATAC | 235 |
| JK669359 | mEgEST272 | (agg)5 | TCTGTCCTCTCCTCCTCTCG | CCACTCCGTGTTCTTGTTCA | 231 |
| GH635922 | mEgEST273 | (cat)5 | TCTTTTCCCTCGTCCTCTAT | AGGACTGCTGAGTGGAGAT | 217 |
| GT119344 | mEgEST274 | (tc)19(ga)7(ag)6 | TGATGGTTCGTTGACATTGC | TTTGTGCACACACCAAGACA | 217 |
| JK669627 | mEgEST275 | (atca)3 | TGCATTTACTCGTGAGCATGT | ACAAATCACGTCCGGTTAGG | 214 |
| CN600275 | mEgEST276 | (ct)7 | TGCCTCATCTTCCATTCCTC | CACACATTTCCCTTCAGAGTC | 183 |
| JK669150 | mEgEST277 | (aat)6 | TGCTGGATCCTTGGCTATTC | TGTTCGCATGGTAGCTTGTC | 162 |
| GT120673 | mEgEST278 | (aagag)3 | TGGACAACAAGAGGCTGTGA | CCTCACCAAATCTTGGGCTA | 204 |
| CN600010 | mEgEST279 | (gtg)5 | TGGGTTTATTTGCCCTTTCA | TGACCATGACCCTCCGTTT | 229 |
| GT119572 | mEgEST280 | (tgc)5 | TGTCAGGCTGTCATTGGAGA | GGGAAGACCAATTGCAAAGA | 176 |
| JK668612 | mEgEST281 | (tttc)3 | TGTGCATTTCATCCTCGGTA | TACCATTCCCTTCTGCTGCT | 181 |
| JK669173 | mEgEST282 | (catg)3 | TTATGGTGGCCTTGTGTTGA | AGAAATGGAGGGAGGGAAGA | 248 |
| GT119446 | mEgEST283 | (tct)6 | TTCAAGTTCCCACCCTCATC | CCTTCACCAGCCTTGAAGAA | 246 |
| CN600415 | mEgEST284 | (ggc)5 | TTCAGAGCGAGCCTAAACCT | CAATATCAGCAATCCCGTCA | 234 |
| JK669064 | mEgEST285 | (ag)6 | TTCGCAAGAGATTTCAGGT | TAGGTTGATTAAGCCAAGGA | 252 |
| JK669628 | mEgEST286 | (ag)14 | TTCTCTTCGCCTTTTCCAGA | GGGAGGAGGAATTCCATTTG | 156 |
| GH637120 | mEgEST287 | (agct)3 | TTGGCTGGAGGTTTAGCTTC | GAACCCATTGCACAAAAACC | 243 |
| CN599543 | mEgEST288 | (ttgt)3 | TTTCACCTGCAACTCATCCA | TTGGCTGAAGGAGCAAGATT | 237 |
| CN599492 | mEgEST290 | (ctctcc)4 | TTTTCGTCGCCACCGATAC | CAACGTCCGCCTTTGGAC | 201 |
| GH637749 | mEgEST291 | (tc)8 | TTTTCTGGTCTACGGAAGC | ACCATTCAGAGAAAGCACAG | 160 |

^1^The 5’ extension CACGACGTTGTAAAACGAC was added to all forward primers; ^2^predicted PCR product sizes; ^3^An annealing temperature of 52 °C and an MgCl_2_ concentration of 0.6 mM was used for the PCR reactions (See Methods section).
